# Supplementary material for: The interactome of intact mitochondria by cross-linking mass spectrometry provides evidence for coexisting respiratory supercomplexes
Source: Mol Cell Proteomics. 2017 Dec 8;17(2):216–32. doi: 10.1074/mcp.RA117.000470 (PMC5795388; doi:10.1074/mcp.RA117.000470)
Supplement: Supplemental Data [file supp_RA117.000470_133922_0_supp_23978_fzffwf.zip › spectra_annotation/mito_DR_spectra_annotation/373-1-6-1-1-1.pdf]

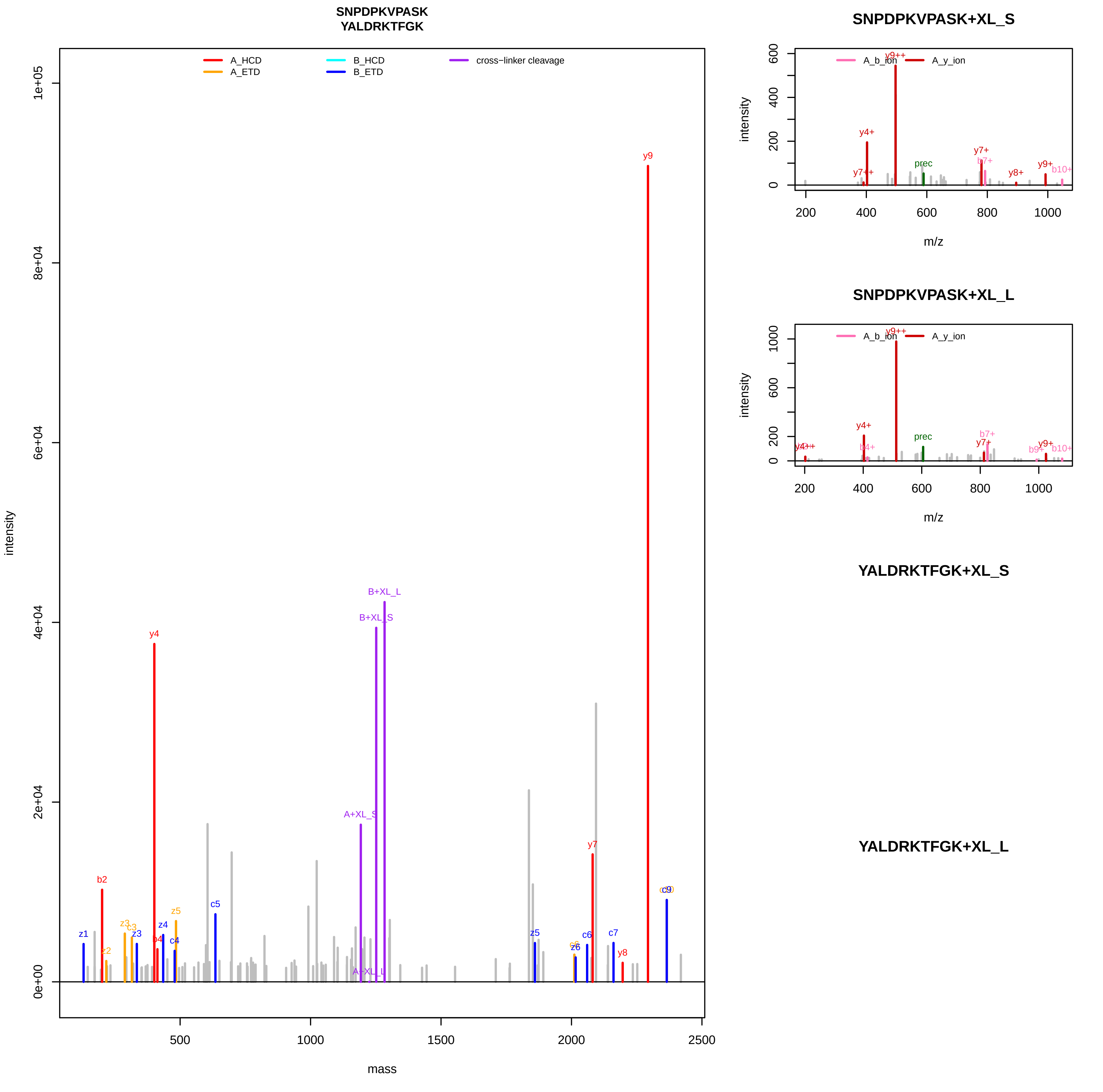
SNPDPKVPASK
YALDRKTFGK
A_HCD
A_ETD
B_HCD
B_ETD
cross−linker cleavage
intensity
mass
y9
y4
b2
y7
y8
B+XL_L
B+XL_S
A+XL_S
A+XL_L
z1
z2
z3
c3
z4
c4
z5
c5
b4
z5
z6
c6
c7
c9
SNPDPKVPASK+XL_S
SNPDPKVPASK+XL_L
A_b_ion
A_y_ion
y9++
y4+
y7++
y7+
b7+
prec
y8+
y9+
b10+
b4+
b9+
m/z
YALDRKTFGK+XL_S
YALDRKTFGK+XL_L
